# What will others think of me? The longitudinal association between trauma-related shame and guilt and psychopathology after a terror attack

**DOI:** 10.1192/bjo.2023.624

**Published:** 2024-01-11

**Authors:** Kristin Alve Glad, Helene Flood Aakvaag, Tore Wentzel-Larsen, Grete Dyb, Siri Thoresen

**Affiliations:** Division for Disasters, Terror and Stress Management, Norwegian Centre for Violence and Traumatic Stress Studies (NKVTS), Oslo, Norway; Division for Disasters, Terror and Stress Management, Norwegian Centre for Violence and Traumatic Stress Studies (NKVTS), Oslo, Norway; and Division for Service Research and Innovation, Centre for Child and Adolescent Mental Health, Eastern and Southern Norway, Oslo, Norway; Division for Disasters, Terror and Stress Management, Norwegian Centre for Violence and Traumatic Stress Studies (NKVTS), Oslo, Norway; and Institute of Clinical Medicine, University of Oslo, Norway; Division for Disasters, Terror and Stress Management, Norwegian Centre for Violence and Traumatic Stress Studies (NKVTS), Oslo, Norway; and Department of Psychology, University of Oslo, Norway

**Keywords:** Mass trauma, shame, guilt, post-traumatic stress disorder, mental health

## Abstract

**Background:**

Trauma-related shame and guilt have been identified as important factors for mental health following interpersonal trauma. For survivors of terror and disasters, however, the role of shame and guilt remains largely unknown.

**Aims:**

To explore the long-term occurrence of trauma-related shame and guilt among survivors of a terror attack, and the potential importance of these emotions for mental health.

**Method:**

A total of 347 survivors (48.7% female, mean age at the time of the attack: 19.25 years, s.d. = 4.40) of the 2011 massacre on Utøya island, Norway, participated in face-to-face, semi-structured interviews. Trauma-related shame and guilt were measured with items from the Shame and Guilt After Trauma Scale at 2.5 and 8.5 years post-terror attack. Post-traumatic reactions and anxiety/depression at 8.5 years post-terror attack were measured with the University of California at Los Angeles PTSD Reaction Index and the Hopkins Symptom Checklist-25, respectively. Associations between trauma-related shame/guilt and post-trauma psychopathology were analysed by multiple linear regressions.

**Results:**

Trauma-related shame and guilt were prevalent among survivors at both 2.5 and 8.5 years post-terror attack. In unadjusted analyses, shame and guilt, at both time points, were significantly associated with post-traumatic stress reactions and anxiety/depression. Shame remained significantly associated with mental health when adjusted for guilt. Both earlier and current shame were uniquely related to mental health.

**Conclusions:**

Trauma-related shame and guilt may be prevalent in survivors of mass trauma several years after the event. Shame, in particular, may play an important role for long-term mental health. Clinicians may find it helpful to explicitly address shame in treatment of mass trauma survivors.

Trauma-related shame and guilt have been found in populations exposed to a variety of potentially traumatic events, including domestic violence, sexual abuse and military trauma.^1–3^ These emotions, particularly shame, seem to be important risk factors for mental health development post-trauma.^4–6^ However, to date, the role of shame and guilt for mental health has not been thoroughly investigated in survivors of mass trauma. In the present study, we explore the occurrence of trauma-related shame and guilt among more than 200 survivors of a mass shooting in Norway, and examine whether these emotions are associated with impaired mental health almost a decade post-trauma.

## Definitions of shame and guilt

Shame can be considered a painful affective experience, typically combined with perceptions that the individual has personal attributes, personality characteristics, or has engaged in behaviours, that others will find unattractive, and that will result in rejection or some kind of humiliation.^7^ Thus, shame is a social emotion, and alerts the individual that their social position is under threat.^7,8^ Alternative definitions emphasise other aspects of shame; for example, that it is a global devaluation of the self.^9,10^ Guilt can be considered ‘an unpleasant feeling with an accompanying belief that one should have felt, thought or acted differently’.^11^ Tangney emphasises that guilt is related to the devaluation of specific behaviours, rather than the devaluation of the global self, as with shame.^12,13^ Previous research has found that shame and guilt often arise after, and in relation to, trauma.^5,14^ One explanation for this relates to the individual's meaning making of the trauma, including perceiving it as entailing an attack on the self or a loss of status or social attractiveness (shame), or that it entails a departure from personal standards, a responsibility for harm to others or a lack of justification for actions taken (guilt).^15^ Studies of trauma-related shame and guilt have tended to focus on violence and sexual abuse, although there is some indication that these reactions may be relevant to mass trauma as well.^16^

## Mass trauma

Mass trauma typically affects large groups of individuals simultaneously, and includes events such as school shootings, major accidents, natural disasters and terror attacks. Mass trauma events affect millions of people around the world, and exposure to such events has been associated with a variety of mental health consequences, including post-traumatic stress disorder (PTSD), major depressive disorder and substance use disorder.^17^ Exposure to a mass trauma may differ from exposure to interpersonal trauma in ways pertinent to shame and guilt. For example, as noted by Aakvaag et al, mass trauma events are generally not secret.^16^ Rather, the massive public attention of mass traumas will often entail that the survivor's social network know about the event.^16^ As such, the public nature of the event partly omits the issue of disclosure, thought to be central to shame.^18^ In contrast to interpersonal trauma (e.g. sexual assault), mass trauma is usually not directed at any one particular individual, and thus potentially stimulates less self-blame and self-devaluation. However, public attention post-trauma may entail aspects that can contribute to shame and guilt. Survivors are often publicly exposed in a vulnerable situation. Although the bulk of public attention may be supportive, criticism of actions or inactions during the event may be voiced. For example, a recent study found that a third of the survivors of the terror attack on Utøya island had received hate speech and/or threats post-terror attack, including blame for the attack, for trying to escape or for the death of their friends.^19^ Although survivors may not be able to hide the fact that the event itself happened to them, they may still conceal certain aspects of the event, such as those that are particularly shameful or guilt-inducing (e.g. not helping someone in need). Thus, there is reason to believe that although mass traumas may differ from other traumas in ways pertinent to shame and guilt, these emotions may still be potent among survivors.

## The relationship between trauma-related shame and guilt and mental health

Recent meta-analyses have supported the important roles of trauma-related shame and guilt for mental health among survivors of interpersonal violence and military veterans.^5,20,21^ However, little is known about trauma-related shame and guilt after mass trauma. For example, in a review on the role of guilt in the development of PTSD, Pugh et al identified 27 relevant studies, of which none concerned survivors of mass trauma.^20^ A later study, using single-item measurements of shame and guilt, identified such emotions to be present in about one out of ten earthquake survivors, with a higher prevalence in participants with probable PTSD.^22^ Another paper of particular interest for the present study, which was not included in the review by Pugh et al, is a long-term follow-up study of survivors of the Piper Alpha oil platform disaster. Ten years after the disaster, Hull et al found that a third of the sample reported current survivor guilt (‘I should not have survived’), and a third reported current performance guilt (‘I should have done better’), which were both associated with post-traumatic stress severity.^23^ This study did not, however, control for shame. As guilt and shame are highly related, both factors should preferably be included to assess each factor's unique association to health.

Regarding the association between shame and PTSD, a review by Saraiya and Lopez-Castro identified 47 studies, of which only one concerned survivors of mass trauma.^21^ This study was a preliminary investigation into shame and guilt in an early phase after the 2011 Utøya terror attack.^16^ Results showed that these emotions occurred in a significant minority of survivors and were associated with concurrent post-traumatic stress reactions (PTSRs). Although this study had some methodological limitations (e.g. a simplistic measure of shame and guilt and a cross-sectional design), the results suggested that shame and guilt might be key for mental health post-trauma. Given the long-term suffering in many victims, it is of pivotal importance to identify drivers of psychopathology among survivors of mass trauma.

## Aims

In this study, we assessed the role of shame and guilt for long-term mental health, using a longitudinal design and a more elaborate measure of trauma-related shame and guilt. Our aims were to explore the occurrence of trauma-related shame and guilt among the survivors of the 2011 Utøya terror attack at 2.5 and 8.5 years post-trauma, and to assess their associations with post-traumatic stress, anxiety and depression at 8.5 years.

## Method

The Utøya Study (2011–2020) is a comprehensive longitudinal interview study designed to determine the mental health development and its determinants among survivors of the terror attack on Utøya island, Norway, in 2011. The study consists of four data collection waves, conducted at 4–5 months (time point 1), 14–15 months (time point 2), 30–32 months (time point 3) and 8.5 years (time point 4) post-terror attack. The current paper uses data from time points 3 and 4, at which time points a more comprehensive measurement of shame and guilt was included in the interview guide.

### The terrorist attack on Utøya island

On 22 July 2011, a lone-acting terrorist committed a mass shooting on the small island of Utøya, a 40-min drive from the capital of Norway, where the youth organisation of the Norwegian Labor Party hosted their annual summer camp. Almost 600 people participated at the camp, mostly adolescents and young adults. The attack lasted approximately 1 h 20 mins, during which the perpetrator shot and killed or wounded those he came across. Sixty-nine people were killed, and many were injured.^24^ The survivors experienced a high level of traumatic exposure, including life-threatening experiences and horrific sensory impressions, and deaths of close friends.

### Participants

In total, 502 individuals survived the massacre on Utøya island and were invited to participate in the Utøya Study. Of these, 347 participated at time point 3 and/or 4 (266 at time point 3, 289 at time point 4 and 208 at both time points). The survivors’ mean age at the time of the terror attack was 19.25 years (s.d. = 4.40, range 13.3–56.7), and 48.7% were female. The vast majority (91.6%) were of Norwegian origin (i.e. one or both parents were born in Norway). When asked to rate how they perceived their financial well-being compared with others, on a scale from 1 to 5 (much poorer, somewhat poorer, similar, somewhat better or much better), 21.1% reported that they perceived themselves as financially disadvantaged (i.e. much or somewhat poorer than others). A more comprehensive description of the participants has been reported elsewhere.^19,25^

### Procedures

All survivors of the terrorist attack on Utøya were invited to participate in the study. Interviews were conducted face to face, with experienced health personnel (mostly psychologists, medical doctors and nurses) acting as interviewers. Participation was based on informed written consent for adolescents aged ≥16 years, and on parental written consent for younger children, in accordance with Norwegian law. Part of the interview guide consisted of a self-report section, which was completed by the respondents with the interviewer beside them and available for questions. If interviewers identified unmet needs among the participants, they were instructed to arrange for assistance (e.g. referral to mental health services). At time point 4, 18 participants were not able to attend a face-to-face interview but responded to the same measures in an online questionnaire. The study was approved by the Regional Committee for Medical and Health Research Ethics in Norway (reference number #2011/1625).

### Measures

#### Trauma-related shame and guilt

Trauma-related shame and guilt were measured at time points 3 and 4, using seven items from the Shame and Guilt After Trauma Scale (SGATS;^16^ three items addressing shame and four items addressing guilt). Individual items are displayed in Table 1. Each item was rated on a 0–2 scale, with the following options: no; yes, a little and yes, a lot. At 2.5 years, post-traumatic shame and guilt were measured with the following instruction: ‘Have you experienced any of the following in relation to 22 July 2011 or events related to 22 July?’. At 8.5 years, current trauma-related shame and guilt were measured with the following instruction: ‘Nowadays: Do you experience any of the following in relation to what happened on Utøya the 22 July?’. Mean scores were calculated (range: 0–2) for individuals if at least half of the items in the score had valid responses on each subscale (i.e. the ‘half rule’). At time point 3, Cronbach's alpha was 0.61 for shame and 0.80 for guilt. At time point 4, Cronbach's alpha was 0.67 for shame and 0.85 for guilt.

**Table 1:** Level of trauma-related shame and guilt reported by the survivors at 2.5 years (*n* = 264–266) and 8.5 years (*n* = 289) post-terror attack

	2.5 years			8.5 years		
	No	Yes, a little	Yes, a lot	No	Yes, a little	Yes, a lot
Shame, *n* (%)						
Worried about what others might think of you after what happened	97 (36.5)	128 (48.1)	41 (15.4)	138 (47.8)	120 (41.5)	31 (10.7)
Tried to conceal what happened, or any part of it	146 (54.9)	92 (34.6)	28 (10.5)	157 (54.3)	101 (34.9)	31 (10.7)
Looked down on yourself after what happened	179 (67.3)	69 (25.9)	18 (6.8)	228 (78.9)	52 (18.0)	9 (3.1)
Guilt, *n* (%)						
Been bothered by thoughts that you should have done something differently to prevent what happened	152 (57.1)	80 (30.1)	34 (12.8)	221 (76.5)	54 (18.7)	14 (4.8)
Been bothered by thoughts that you should have done something differently while it was happening	109 (41.1)	108 (40.8)	48 (18.1)	183 (63.3)	83 (28.7)	23 (8.0)
Felt that you did anything wrong	198 (74.4)	57 (21.4)	11 (4.1)	249 (86.2)	34 (11.8)	6 (2.1)
Experienced any feelings of guilt about any part of what happened	154 (57.9)	84 (31.6)	28 (10.5)	214 (74.0)	60 (20.8)	15 (5.2)

The wording was identical at the two time points, the observation period differed (i.e. at 2.5 years the participants were asked to report shame and guilt experienced at any time after the trauma, whereas at 8.5 years they reported current emotions). Thus, the observed prevalences are not directly comparable over time.

#### Post-traumatic stress reactions (PTSR)

PTSR at time point 4 were measured with the University of California at Los Angeles PTSD Reaction Index (UCLA PTSD-RI).^26,27^ Participants were asked to indicate whether symptoms had occurred within the previous month. The index was customized in collaboration with the authors of the original instrument in preparation for the first data collection of the Utøya Study in 2011, to cover all diagnostic criteria in the DSM-5. Thus, the measure comprises 20 items, with subscales on the four diagnostic criteria: re-experiencing (five items), avoidance (two items), negative alterations in cognitions and mood (seven items), and arousal and reactivity (six items). All items are explicitly related to the attack, and responses were endorsed on a five-point scale, ranging from 0 (never) to 4 (almost all the time). We used the UCLA PTSD-RI as a continuous score in the regression analyses, and the mean score was calculated with all 20 items. Missing data were handled with the half rule. The Cronbach's alpha of the total scale was 0.92 (time point 4).

After the introduction of the DSM-5, the PTSD diagnosis includes the D criterion of ‘Negative thoughts or feelings that began or worsened after the trauma’. Such negative thoughts or feelings may include blame of self or others or negative thoughts about oneself or the world. Thus, there may be some conceptual overlap between our predictors (guilt and shame) and the outcome (PTSR). To investigate whether this overlap contributed to the results, we conducted sensitivity analyses where the mean score of the UCLA PTSD-RI was calculated based only on the other three criteria (re-experiencing, avoidance and arousal), using the ‘half rule’ to handle missing data. The Cronbach's alpha was 0.88.

#### Symptoms of depression and anxiety

Symptoms of depression and anxiety were measured with an eight-item version of the Hopkins Symptom Checklist-25 (SCL-8).^28^ Respondents indicated how bothered they had been by each symptom in the past 2 weeks on a scale from 1 (not at all bothered) to 4 (very much bothered). Previously, short versions of the SCL used in Norwegian population surveys have shown high correlation with the 25-item scale and good psychometric properties.^29^ Missing data were handled with the 'half rule'. The Cronbach's alpha of the total scale was 0.90 at time point 4.

#### Potential confounders

Traumatic exposure was measured by a 13-item yes or no checklist, covering items such as ‘saw the terrorist or heard his voice’ and ‘saw dead bodies’. A sum score was constructed as a count of the number of ‘yes’ answers (range 0–13).^25^ Sociodemographic variables included gender, age, national origin (Norwegian versus non-Norwegian) and financial status (poor versus medium/high).

### Statistical analysis

In total, 347 survivors participated in the Utøya Study at time points 3 and/or 4 (266 at time point 3, 289 at time point 4 and 208 at both time points). In the descriptive analyses, we included everyone who had participated at each time point. To explore the occurrence of trauma-related shame and guilt reported at time points 3 and 4, we dichotomised the variables (0 *v.* 1–2). Of the 208 survivors who participated at both time points 3 and 4, we had complete data on 206, and they formed the sample for the correlation and regression analyses. First, we did correlation analyses to explore the associations between the main variables. Then, univariable linear regression models were run to investigate the associations between trauma-related shame and guilt at 2.5 and 8.5 years post-trauma and psychopathology at 8.5 years. Subsequently, PTSR and anxiety/depression at time point 4 served as the continuous outcomes of multiple linear regression models, estimating the impact of trauma-related shame and guilt on psychopathology at 8.5 years post-terror attack. For each dependent variable, we ran two models. In model 1, analyses included trauma-related shame and guilt at time points 3 and 4. In model 2, analyses additionally included sociodemographic characteristics (i.e. gender, age at time of attack, ethnicity) and trauma exposure. Finally, we conducted sensitivity analysis to compare the association between trauma-related shame and guilt at both time points with PTSR without the D criterion (gender, age at time of attack, ethnicity and trauma exposure were included in this analysis). Bias-corrected and accelerated confidence intervals for differences with and without inclusion of the D criterion were computed based on 10 000 bootstrap replications. Analyses were conducted in R software in Windows (R may be downloaded from https://cloud.r-project.org/ subsites ‘Download R for Windows’ and ‘base’), version 4.1.2 (the R Foundation for Statistical Computing, Vienna, Austria), with the R package boot for bootstrap analyses.

## Results

Overall, feelings of shame and guilt were common at both measurement times. At 2.5 years post-terror attack, 78% of the survivors reported that they had experienced at least some trauma-related shame (i.e. ‘yes, a bit’ or ‘yes, a lot’ to at least one of the three shame items), whereas 71% reported that they had experienced trauma-related guilt (i.e. ‘yes, a bit’ or ‘yes, a lot’ to at least one of the four guilt items). At 8.5 years post-terror attack, 68% of the participants reported that they currently experienced at least some trauma-related shame, whereas 45% reported at least some feelings of guilt. The most prevalently reported shame item, at both time points, was being worried about what other people might think about them after the attack. The most prevalently reported guilt item, at both time points, was bothersome thoughts about something they could have done differently during the attack (Table 1).

We found a moderate correlation between trauma-related shame and guilt, and between each of these emotions at the two time points (Table 2).

**Table 2:** Correlations between trauma-related shame and guilt and psychopathology post-terror attack (*n* = 206)

		1	2	3	4	5	6
1	Time point 3 shame	1.000					
2	Time point 3 guilt	0.466	1.000				
3	Time point 4 shame	0.496	0.409	1.000			
4	Time point 4 guilt	0.436	0.576	0.676	1.000		
5	Post-traumatic stress reactions	0.471	0.417	0.637	0.562	1.000	
6	Anxiety/depression	0.385	0.275	0.515	0.388	0.800	1.000

*P* for all correlations is <0.001.

In the unadjusted analyses, shame and guilt at both time points were associated with PTSR at 8.5 years after the terror attack (Table 3). When adjusting for trauma-related shame and guilt at both time points (model 1), shame at time points 3 and 4, as well as guilt at time point 4, remained significantly associated with PTSR. Highly similar results were observed when adjusting for sociodemographic characteristics and trauma exposure (model 2). Results from the sensitivity analysis indicated that there was no evidence for differences in the association between trauma-related shame/guilt and PTSR when the items of the UCLA PTSD-RI including the D criterion were removed (results not shown).

**Table 3:** Linear regression analysis displaying associations between post-traumatic stress reactions and trauma-related shame and guilt post-terror attack (*n* = 206)

	Unadjusted		Model 1		Model 2	
	Coefficient^a^ (95% CI)	*P-*value	Coefficient (95% CI)	*P-*value	Coefficient (95% CI)	*P-*value
Time point 3 shame	0.33 (0.25–0.42)	<0.001	0.11 (0.02–0.20)	0.014	0.10 (0.01–0.19)	0.031
Time point 3 guilt	0.27 (0.19–0.36)	<0.001	0.05 (−0.03 to 0.14)	0.242	0.04 (−0.05 to 0.13)	0.356
Time point 4 shame	0.50 (0.42–0.59)	<0.001	0.32 (0.21–0.44)	<0.001	0.32 (0.21–0.44)	<0.001
Time point 4 guilt	0.43 (0.35–0.52)	<0.001	0.13 (0.01–0.25)	0.031	0.12 (0.00^b^–0.244)	0.045

Model 1: all trauma-related shame and guilt variables are included in the regression analysis. Model 2: also adjusted for gender, age at time of attack, ethnicity and trauma exposure. Time point 3 was 2.5 years post-terror attack, time point 4 was 8.5 years post-terror attack.

a. Regression coefficient.

b. Result was larger than 0.00.

As with PTSR, shame at both time points were uniquely associated with anxiety/depression (Table 4). Guilt, however, was not.

**Table 4:** Linear regression analysis displaying associations between anxiety/depression and trauma-related shame and guilt post-terror attack (*n* = 206)

	Unadjusted		Model 1		Model 2	
	Coefficient^a^ (95% CI)	*P-*value	Coefficient (95% CI)	*P-*value	Coefficient (95% CI)	*P-*value
Time point 3 shame	0.34 (0.23–0.46)	<0.001	0.15 (0.02–0.27)	0.026	0.13 (0.00^b^–0.27)	0.043
Time point 3 guilt	0.23 (0.12–0.34)	<0.001	0.01 (−0.12 to 0.14)	0.862	0.00 (−0.12 to 0.13)	0.948
Time point 4 shame	0.52 (0.40–0.64)	<0.001	0.41 (0.24–0.57)	<0.001	0.40 (0.23–0.57)	<0.001
Time point 4 guilt	0.38 (0.26–0.51)	<0.001	0.04 (−0.14 to 0.21)	0.687	0.03 (−0.14 to 0.21)	0.727

Model 1: all trauma-related shame and guilt variables are included in the regression analysis. Model 2: adjusted for gender, age at time of attack, ethnicity and trauma exposure. Time point 3 was 2.5 years post-terror attack, time point 4 was 8.5 years post-terror attack.

a. Regression coefficient.

b. Result was larger than 0.00.

## Discussion

In this study, we document a high occurrence of trauma-related shame and guilt among survivors of a mass shooting. Further, our findings demonstrate the importance of these emotions for mental health almost a decade after the attack.

We found that most participants reported trauma-related shame and/or guilt at some time point after the terror attack on Utøya island. Even 8.5 years after the terror attack, more than half of the participants reported current feelings of trauma-related shame, and almost half reported trauma-related guilt, which suggest that these emotions are common and long-lasting among survivors of mass trauma.

The most frequently reported aspect of shame at both 2.5 and 8.5 years post-terror attack (>50%), was being worried about what other people might think about them after the attack. Many participants also reported that they were trying to conceal what happened or some part of it. Both these aspects underscore the social/interpersonal component of shame. Shame as a prevalent response to mass trauma has not previously been investigated,^21^ perhaps because such traumas are not viewed as particularly stigmatising. However, as Lee et al have pointed out, a traumatic event can evoke intense feelings of shame despite the fact that, at face value, it may not be understood as a ‘shaming event’.^15^ They argue that shame can arise as a result of particular aspects of the traumatic event (primary) or later on, because of signs of weakness or not being able to cope (secondary). Whereas this may be true for a variety of traumatic events, survivors of mass trauma are more easily identifiable, and concealment or disclosure is not always within the survivor's control (e.g. media exposure displaying victims in vulnerable situations).

Some form of guilt was reported by a majority of the participants at 2.5 years after the event, and by almost half of participants at 8.5 years. The most frequently reported aspect of guilt at both time points was bothersome thoughts about something the survivor could have done differently during the attack. This is in line with the finding by Hull et al, where a third of the sample reported current performance guilt (‘I should have done better’) 10 years after an oil platform disaster.^23^ In mass trauma events there is often a considerable time period before victims are in safety. As noted by Aakvaag et al, in the midst of the disaster, survivors encounter numerous choices (e.g. including whether to run or to hide, to stay in a group or flee alone).^16^ Decisions on how to act are made quickly, based on limited information and may have dramatic consequences for themselves and other people, which might also explain the high occurrence of this aspect of guilt such a long time post-trauma.

### Trauma-related shame and mental health

Shame at both time points remained significantly associated with mental health at 8.5 years post-terror attack when adjusting for guilt. This is in line with previous literature, which underscores the importance of shame for mental health.^6^ Several potential mechanisms explaining how shame is implicated in psychopathology have been suggested. For example, shame is often conceptualised as a social emotion,^30^ whose function is to warn the individual that the social self is under threat.^7,8^ Scholars have argued that the behavioural correlates of shame (including hiding, withdrawal and submissiveness) signify attempts to protect oneself against rejection or humiliation.^30^ These behaviours, however, may block the individual's potential for social support, erode social bonds and, over time, result in loneliness, all of which render the individual vulnerable to mental health problems.^31^ In fact, loneliness has been found to mediate the relationship between shame and anxiety/depression.^32^ It is also possible that trauma-related shame fuels specific maladaptive cognitions, which are well-documented precursors for the development of PTSD.^33^ For example, survivors might have cognitions related to their own competency and ability to deal with adversity, such as ‘I am weak’ or ‘I am not able to protect myself’. Further research on the mechanisms involved in the strong link between shame and mental health post-trauma is warranted.

Notably, we found a significant association between current trauma-related shame and psychopathology at 8.5 years after the attack, even when earlier trauma-related shame was adjusted for. It is possible that both early and later shame can contribute to long-term psychopathology. Thus, trauma-related shame can be viewed as a dynamic factor that may vary in content and intensity over time. However, more research is needed. For example, future research could investigate the development of trauma-related shame over time.

### Trauma-related guilt and mental health

Our results indicated that current guilt, when adjusted for shame, had a small-to-moderate association with PTSR, but not with anxiety and depression. The strength of the association between guilt and both types of psychopathology was largely reduced when adjusting for shame. This indicates that much of the contribution of guilt to mental health problems may rest on its association with shame. However, in line with Shi et al, we conclude that the unique role of guilt in PTSD cannot be ruled out.^6^ Our results underscore the importance of accounting for guilt when studying shame, and *vice versa*, as previously stressed by Pugh et al.^20^

### Study strengths and limitations

Certain limitations should be taken into account when interpreting our findings. First, at time point 3, participants were asked to report trauma-related shame and guilt over the period of 2.5 years that had passed since the attack. Thus, these responses may have been affected by recall bias. Second, to avoid overburdening the participants, only seven of the nine shame and guilt (SGATS) items were included (four items measuring guilt, three measuring shame), which probably contributed to the low internal consistency for the trauma-related shame measure. Third, a more comprehensive measure might have captured the multifaceted experience of post-traumatic shame better. Finally, the nature of the traumatic event the study participants had been exposed to (i.e. significant life threat in a human-made mass trauma followed by intense media attention), and their developmental stage at the time of the attack (i.e. mainly youth and young adults), may limit the generalisability of the findings. Study strengths include the longitudinal design, the use of face-to-face interviews, the very low levels of missing data and the relatively high response rate.

### Implications and future research

The fact that most participants reported trauma-related shame and/or guilt at some time point after the terror attack on Utøya island suggests that these emotions are common among survivors of mass trauma. This is important knowledge for clinicians. Further, the results from this study indicate that trauma-related shame may be important to the mental health development of survivors of mass trauma, and it may be beneficial to address shame in treatment of post-traumatic reactions in this population. Of note, as pointed out by López-Castro et al, a therapeutic focus on shame may cut across diagnostic lines.^14^ For example, a reduction in maladaptive shame among trauma survivors is likely to be beneficial not only with regards to symptoms of PTSD, but also comorbid psychopathology, such as depression.^34^ That said, there is a need for more research. Longitudinal studies with several time points and earlier data collections are warranted to explore the sequential development of shame, guilt and mental health problems, including their potential mutual influence. Future studies are also needed to explore in more detail what people exposed to mass trauma feel ashamed of, whether the object of shame feelings changes over time and if shame is influenced by (negative) responses from other people. Further, we need better insight into the mechanisms involved in the shame–psychopathology link, particularly related to the importance of social relationships and maladaptive appraisals.

On a final note, although individual treatment is of vital importance, community responses to trauma should not be neglected. Mass trauma can affect the social fabric of communities and disrupt social networks, and community responses can potentially reach out to a large population of victims.^35,36^ In a previous paper, we have described a community perspective as applied to research on and interventions for those directly affected by the terror attack on Utøya.^36^ We have noted, for example, that mental health may be affected by media attention and media participation,^37^ and experiences with hate speech.^19^ Further research is necessary, however, to assess the efficiency of community responses and reveal the potential links between community responses, shame, guilt and mental health.

## Data Availability

The data cannot be made available because of the personal and sensitive content of participants’ experiences.
